# The Role of TPA I/D and PAI-1 4G/5G Polymorphisms in Multiple Sclerosis

**DOI:** 10.1155/2014/362708

**Published:** 2014-04-16

**Authors:** Maja Živković, Nada Starčević Čizmarević, Luca Lovrečić, Inge Klupka-Sarić, Aleksandra Stanković, Iva Gašparović, Polona Lavtar, Evica Dinčić, Ljiljana Stojković, Gorazd Rudolf, Saša Šega Jazbec, Olivio Perković, Osman Sinanović, Juraj Sepčić, Miljenko Kapović, Borut Peterlin, Smiljana Ristić

**Affiliations:** ^1^Laboratory for Radiobiology and Molecular Genetics, “Vinča” Institute of Nuclear Sciences, University of Belgrade, 11000 Belgrade, Serbia; ^2^Department of Biology and Medical Genetics, School of Medicine, University of Rijeka, 51000 Rijeka, Croatia; ^3^Clinical Institute of Medical Genetics, University Medical Centre, 1000 Ljubljana, Slovenia; ^4^Department of Neurology, School of Medicine, University of Mostar, 88000 Mostar, Bosnia and Herzegovina; ^5^Department of Neurology, Clinical Hospital Center Rijeka, 51000 Rijeka, Croatia; ^6^Neurology Clinic, Military Medical Academy, 11000 Belgrade, Serbia; ^7^Department of Neurology, University Medical Centre, 1000 Ljubljana, Slovenia; ^8^Department of Neurology, School of Medicine, University of Tuzla, 75000 Tuzla, Bosnia and Herzegovina; ^9^Postgraduate Studies, School of Medicine, University of Rijeka, 51000 Rijeka, Croatia

## Abstract

*Background*. Previous studies have shown impaired fibrinolysis in multiple sclerosis (MS) and implicated extracellular proteolytic enzymes as important factors in demyelinating neuroinflammatory disorders. Tissue-type plasminogen activator (t-PA) and its inhibitor (PAI-1) are key molecules in both fibrinolysis and extracellular proteolysis. In the present study, an association of the TPA Alu I/D and PAI-1 4G/5G polymorphisms with MS was analyzed within the Genomic Network for Multiple Sclerosis (GENoMS). *Methods*. The GENoMS includes four populations (Croatian, Slovenian, Serbian, and Bosnian and Herzegovinian) sharing the same geographic location and a similar ethnic background. A total of 885 patients and 656 ethnically matched healthy blood donors with no history of MS in their families were genotyped using PCR-RFLP. *Results*. TPA DD homozygosity was protective (OR = 0.79, 95% CI 0.63–0.99, *P* = 0.037) and PAI 5G5G was a risk factor for MS (OR = 1.30, 95% CI 1.01–1.66, *P* = 0.038). A significant effect of the genotype/carrier combination was detected in 5G5G/I carriers (OR = 1.39 95% CI 1.06–1.82, *P* = 0.017). *Conclusions*. We found a significantly harmful effect of the combination of the PAI-1 5G/5G genotype and TPA I allele on MS susceptibility, which indicates the importance of gene-gene interactions in complex diseases such as MS.

## 1. Introduction

Multiple sclerosis (MS) is a complex inflammatory demyelinating disease of the central nervous system (CNS) with both genetic and environmental contributing factors. The underlying cause of the disease is thought to be the cumulative effect of multiple genetic variants throughout the genome, each making a modest contribution to the onset and progression of the disease. However, despite great progress in detecting risk loci for MS using high-throughput technologies over the last decade [1], much remains to be discovered about the genetics of MS [2].

In recent years, increasing evidence has pointed to the potential role of fibrinolysis in the pathogenesis of MS. Specifically, the characteristic inflammation, focal demyelination, and axonal degeneration in MS occur after disruption of the blood-brain barrier (BBB) and entry of serum proteins, including fibrinogen, into the CNS [3]. Extracellular proteolysis represents a potent and irreversible mechanism modulating the extracellular matrix and tissue remodeling, which can affect the breakdown of the BBB. Extracellular proteolytic enzymes have been implicated as important factors in demyelinating neuroinflammatory disorders such as MS [4]. The enzymes of the plasminogen activators/plasmin (PA) system are involved in both fibrinolysis and extracellular proteolyses.

The key molecules in the PA system are tissue-type plasminogen activator (t-PA) and its inhibitor (PAI-1). Due to the formation of t-PA and inhibitor (e.g., PAI) complexes, the fibrinolytic potential in demyelinating MS lesions is greatly diminished [5]. The limited availability of t-PA because of the formation of the t-PA/PAI-1 complexes is assumed to reduce the ability of t-PA receptors to produce plasmin, which further diminishes the fibrinolytic capacity in MS lesions, possibly resulting in increased axonal fibrin deposition and neurodegeneration [6, 7]. On the other hand, it may represent a mechanism to remove fibrin deposits, which are then cleared through internalization by macrophages.

In experimental autoimmune encephalomyelitis (EAE), deletion of the TPA gene results in an earlier appearance and more severe form of disease associated with high levels of PAI-1 and inefficient fibrin removal [7]. Recent studies have shown that the PAI-1^−^ knockout (PAI-1^−/−^) mice have reduced disease incidence, a milder clinical picture, lack of clinical relapse, and reduced neuroinflammation, as well as less axonal damage than wild-type (wt) mice [8].

An Alu-repeat insertion/deletion (I/D) in intron 8 of the TPA gene and the 4G/5G polymorphism in the 5′-untranslated region at −675 of the PAI-1 gene are known, as are differences in the productivity of these proteins between genotypes. The t-PA release rates were markedly higher in subjects homozygous for the I allele [9], whereas individual homozygous for the 4G allele has a 25% higher plasma PAI-1 concentration than those with the 5G5G genotype [10].

To date, only two studies have investigated the correlation between these polymorphisms and MS [11, 12].

Because of technical limitations, the insertion/deletion polymorphism could not be analyzed in recent GWAS studies.

The aim of the present study was to investigate the role of the TPA I/D and PAI-1 4G/5G polymorphisms in a new MS study population and perform a meta-analysis of previous studies [11, 12]. This study was conducted within the Genomic Network for Multiple Sclerosis (GENoMS), which includes four populations (Croatian, Slovenian, Serbian, and Bosnian and Herzegovinian [BH]) that share the same geographic location and a similar ethnic background, a Slavic origin.

## 2. Materials and Methods

### 2.1. Subjects

Cohorts of MS patients collected in Serbia and BH were included in the study. All patients fulfilled the criteria for clinically definite MS [13] and the course of the disease was classified based on clinical data [14]. Patients with relapsing-remitting (RR) or a secondary progressive (SP) course were grouped as bout-onset and analyzed separately from patients with the primary progressive (PP) form. Disease severity was estimated using the Multiple Sclerosis Severity Score (MSSS) [15], which corrects the Expanded Disability Status Scale (EDSS) [16] for disease duration.

A total of 382 Serbian patients (141 males and 241 females) were recruited from the Department of Neurology of the Military Medical Academy (MMA), including 372 (97.4%) patients with bout-onset MS and 10 (2.6%) with PPMS. The mean age at onset was 29.2 ± 7.0 years and the mean MSSS was 5.38 ± 2.52. The Serbian controls were 318 healthy volunteers without a history of MS in their families and of the same ethnic origin as the MS patients.

The BH dataset consisted of 170 patients (50 males and 120 females) recruited consecutively from the Department of Neurology, School of Medicine, University of Mostar and Department of Neurology, School of Medicine, University of Tuzla, and 170 healthy volunteers of matched ethnicity (Bosnians, Croatians, Serbians). Among the patients, 160 (94.1%) had bout-onset MS and 10 (5.9%) had PPMS. The average age at onset was 30.8 ± 8.0 years and the mean MSSS was 4.79 ± 2.81.

For pooled analyses, we included the Croatian and Slovenian subjects, which were the study groups used in a recently published study [12]. The 333 MS patients (94 males, 239 females), including 308 (92.5%) with bout-onset MS and 25 (7.5%) with PPMS, had a mean age at onset of 29.4 ± 8.9 years and mean MSSS of 4.94 ± 2.73. A total of 368 ethnically matched healthy blood donors with no history of MS in their families represented the control group.

Table 1 provides a description of the MS groups included in the study.

### 2.2. Genotyping

Genomic DNA was extracted from whole blood using standard procedures. Genotyping was performed in the Molecular Genetics Laboratory (Department of Biology and Medical Genetics, School of Medicine, Rijeka) using PCR (for the TPA Alu I/D polymorphism) or PCR-RFLP (for the PAI-1 4G/5G polymorphism) as described previously [9, 17].

### 2.3. Statistical Analysis

Statistical analyses were carried out using Statistical Software package for Windows 7.1 (StatSoft, Inc.). Differences in the allele and genotype frequency distribution between the studied groups, as well as deviation from Hardy-Weinberg equilibrium, were estimated by the chi-square (*χ* ^2^) test. A logistic regression analysis expressed in terms of an adjusted odds ratio (OR), and 95% confidence interval (CI) was used as a measure of the strength of the association between the TPA I/D and PAI-1 4G/5G polymorphisms and susceptibility to MS.

To assess the relationship between studied polymorphisms and MS, we performed a meta-analysis of the four datasets, comprising Serbian, BH, Croatian, Slovenian [12], and Finnish subjects [11]. Subjects were classified by diagnostic category (patients and controls) and number of events (the presence of the PAI-1 5G5G genotype or the presence of the PAI-1 5G5G/TPA I carrier combination). The strength of the association was summarized using the OR in which PAI-1 5G5G homozygosity or PAI-1 5G5G/TPA I carriership was assigned as a risk factor for MS. Each of the cohorts was analyzed separately, and the significance of the pooled ORs was determined using the *z*-test. The heterogeneity of the group of ORs was assessed using the *χ* ^2^-based *Q*-statistic test. Meta-analyses were conducted using Comprehensive Meta-Analysis software version 2.0 (Biostat International, Inc.).

Analysis of variance (ANOVA) and appropriate post hoc tests were used to test the relationships between the polymorphisms and continuous variables. In all of the tests, differences with two-tailed *P* ≤ 0.05 were considered significant. Multivariate regression analysis was performed to assess the influence of genetic and clinical parameters on disease onset, clinical course, and MS progression.

### 2.4. Ethics Statement

The study was approved by the respective local ethics committees in all countries participating in the study: Ethics Committee of the MMA, Serbia (reference number: 29/2-05), Ethics committee of the Clinical Hospital Mostar (reference number: 3590/07), the Ethics committee of the University Clinical Hospital Tuzla, Bosnia, and Herzegovina (reference number: 01/3-37-4-7683/07), Ethics committee at School of Medicine at University of Rijeka, Croatia (reference number: 641-01/06-01/66), and the Slovenian National medical ethics committee (reference number: 90/08/12). All participants signed a written informed consent. For those patients who have had a compromised capacity to consent (Folstein Mini-Mental State Examination (MMSE) score below 20) informed consent was signed by the nearest relative or guardian.

## 3. Results

### 3.1. TPA I/D and PAI-1 4G/5G Genotypes and Alleles in MS Patients and Controls

The Serbian and BH case-control groups were analyzed separately and also pooled with Croatian and Slovenian samples [12] following the demonstration of a lack of significant heterogeneity. The distributions of allele and genotype frequencies are provided in Table 2. The genotype distributions among MS patients and controls were compatible with Hardy-Weinberg expectations for both the Serbian and BH cohorts. In the Serbian cohort, PAI-1 5G5G homozygosity (OR = 1.54, 95% CI 1.04–2.28, *P* = 0.032) was positively associated with MS, and 4G5G heterozygosity (OR = 0.68, 95% CI 0.50–0.92, *P* = 0.012) was negatively associated with MS compared to controls. The distribution of the TPA I/D polymorphism was not significantly different in patients compared to controls in the Serbian group. No significant differences were found in the genotype and allele frequency distribution for the polymorphisms in BH patients and controls.

### 3.2. TPA I/D and PAI-1 4G/5G Genotypes in Pooled MS Samples and Meta-Analysis

Comparisons of allele and genotype frequencies between pooled MS patients and controls (Serbian, BH, Croatian, and Slovenian, Table 2) did not reveal significant associations, but we found a trend towards a decreased risk of MS in patients with the TPA DD genotype (OR = 0.80, 95% CI 0.65–1.00, *P* = 0.050) and an increased risk of MS in patients with the PAI-1 5G5G genotype (OR = 1.27, 95% CI 0.97–1.61, *P* = 0.054).

**Figure 1:**
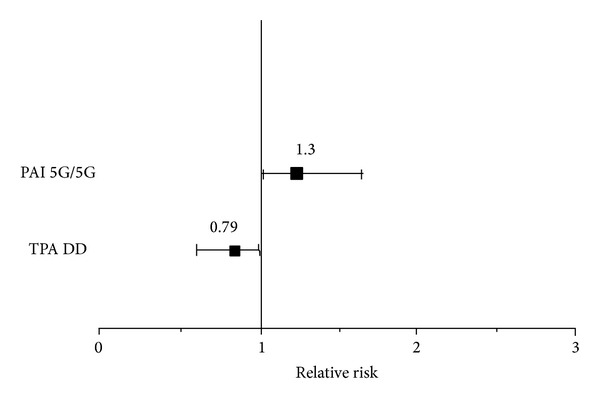
The separate effects of the PAI-1 5G/5G and TPA D/D genotypes on MS susceptibility in a pooled sample of 885 MS patients. Data are presented as the OR adjusted for gender. The error bars represent the 95% CI. TPA D/D, OR = 0.80, 95% CI 0.65–1.00, *P* = 0.050; PAI-1 5G/5G, OR= 1.27, 95% CI 0.97–1.61, *P* = 0.054.

**Figure 2:**
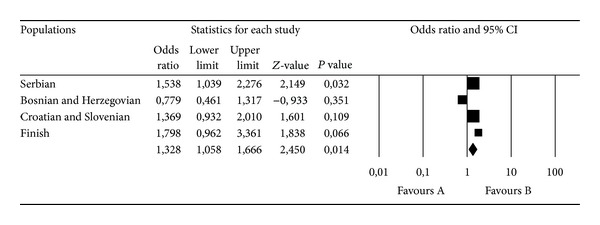
Meta-analysis of the four case-control studies of MS and the PAI-1 4G/5G polymorphism. Comparisons were made between the 5G5G and 4G4G+4G5G genotypes. Summary ORs and respective 95% CIs were calculated using the fixed-effect model.

Although the crude association of the TPA and PAI-1 polymorphisms with MS in a pooled sample (Serbian, BH, Croatian, and Slovenian) did not reveal a significant association after adjusting the ORs for gender, the TPA DD genotype was shown to be significantly protective (OR = 0.79, 95% CI 0.63–0.99, *P* = 0.037) and the PAI-1 5G5G genotype was shown to be a significant risk factor (OR = 1.30, 95% CI 1.01–1.66, *P* = 0.038) for MS (Figure 1). The meta-analysis, which also included the results from a Finnish study [11], confirmed the significant risk for MS among PAI-1 5G5G homozygotes (OR = 1.33, 95% CI 1.06–1.67, *z* = 2.450, *P* = 0.014, *P*_heterogeneity_ = 0.143; Figure 2) compared to the carriers of the 4G allele.

### 3.3. Combination of TPA I/D and PAI-1 4G/5G Genotypes in Pooled MS Samples and Meta-Analysis

**Figure 3:**
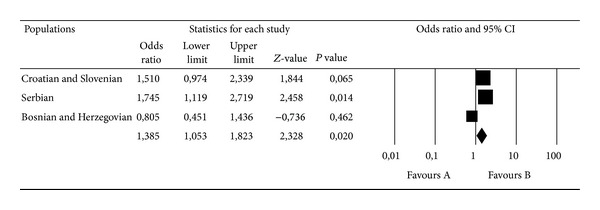
Meta-analysis of the three case-control studies of MS and the PAI-1 4G/5G and TPA I/D polymorphisms. Comparisons were made between the PAI-1 5G5G/TPA I carriers and other genotype/carrier combinations. Summary ORs and respective 95% CIs were calculated using the fixed-effect model.

We analyzed the genotype combinations and/or allele carriership for the TPA I/D and PAI-1 4G/5G polymorphisms in a pooled study sample. The frequency analysis of these combinations is shown in Table 3. The only significant effect of the genotype/allele carrier combination was detected in 5G5G/I carriers (both of these polymorphisms separately carry a significant risk for MS), with an even higher and more significant OR for MS than in the separate analysis (OR = 1.39 95% CI 1.06–1.82, *P* = 0.017). The meta-analysis confirmed this result (OR = 1.38, 95% CI 1.05–1.82, *z* = 2.328, *P* = 0.020, *P*_heterogeneity_ = 0.102; Figure 3). We did not find a significant difference in the distribution of the genotypes and alleles of the tested genes after stratifying by gender.

### 3.4. TPA I/D and PAI-1 4G/5G Genotypes and Clinical Characteristics of MS

We did not find a significant association between the separate or combined effects of the TPA I/D or PAI-1 4G/5G genotypes and disease course (bout onset versus PP), age at disease onset, or MSSS in the Serbian and BH patient samples separately, or in a pooled sample of MS patients (data not shown).

## 4. Discussion

In this study, we analyzed the influence of the TPA I/D and PAI-1 4G/5G polymorphisms on MS. We found a significant, but opposite, influence of the rare TPA and PAI genotypes on MS susceptibility. The TPA D/D genotype had a protective effect, whereas PAI 5G/5G was a risk factor for MS. The meta-analysis for the PAI-1 polymorphism, which included the previously published results [11], confirmed that 5G/5G represents a significant risk factor for MS.

The investigated polymorphisms have been investigated predominantly in vascular disorders [18], rather than diseases such as MS. The single alleles (e.g., 4G) have been found to have opposite effects in cardiac disease (increased risk) compared to brain ischemia (protective effect).

The 4G allele has been linked to higher serum levels of PAI-1 [10]. Recent data suggest a functional importance of the PAI-1 4G/5G polymorphism, which could have a neuroprotective effect through increased astrocytic expression of PAI-1 [19]. Haplotypes containing the PAI-1 4G allele have been associated with higher transcriptional activity in astrocytes compared to haplotypes containing 5G [19]. In this study, the 5G5G genotype exhibited a 1.3-times higher risk for MS susceptibility compared to 4G carriers. In a previous study of Finnish women with MS [11], the 5G5G genotype, which is linked to lower plasma levels of PAI-1 [10], was associated with an increased risk of MS. These results show a consistency in the neuroprotective role of sufficient PAI-1 in the brain through allele-specific regulation of its presence. In addition, the PAI-1 gene is located in the MS susceptibility locus on chromosome 7q21-22 [20]. Only two studies previously looked at the association with MS [11, 12], and GWAS lack data on insertion/deletion polymorphisms, such as the two investigated here.

In the brain, PAI-1 is produced predominantly by astrocytes [21], whereas its main function is to inhibit t-PA [22]. After injury, an excessive amount of t-PA is released into the extracellular space of the brain, which can trigger both neuronal degeneration [23] and disruption of the BBB [24]. Thus, PAI-1 is needed to reduce the deleterious effects of excessive t-PA activity. An increase in brain damage has been shown in PAI-1-deficient mice [25].

In the mouse model of MS, EAE, t-PA has been suggested to have a dual effect. TPA knockout mice exhibit delayed onset of EAE, but the symptoms were more severe and recovery was reduced [26]. This finding suggests that t-PA could have deleterious effects on the initiation of EAE. We found a borderline significance for the protective effect of the TPA D/D genotype in MS patients. The net release rate of t-PA in vivo is significantly higher in the presence of the TPA I/I genotype compared to carriers of the D allele [9].

The increase in plasma t-PA is related to the appearance of EAE symptoms in rats [27]. In the brain, t-PA is localized in the endothelium within regions of neuroinflammation. Monocyte diapedesis through the BBB depends on t-PA activity, which can induce the breakdown of the tight-junction protein occludin, leading to an increase in the permeability of the interface between the blood and the brain, the neurovascular unit (NVU), and subsequent monocyte infiltration into the CNS [28].

Fibrinolysis is generally regulated by the balance between plasminogen activators and inhibitors. Although t-PA and PAI-1 are not the only players in this process, they are among the most important, and the combination genotype is associated with higher PAI-1 and lower t-PA levels, which could be protective against the deleterious effect of t-PA on BBB disruption and lymphocyte infiltration in the brain. The 4G/4G D/D combination, which was not common among MS patients (6.6%) or controls (8.3%), had the protective OR, but was not significant. We found a significantly harmful effect of the combination of the PAI-1 5G/5G genotype and TPA I allele on MS susceptibility. A reasonable explanation could be that the potentially higher t-PA levels and lower PAI-1 levels in carriers of the 5G/5G and I alleles favor the proteolytic function of t-PA. We did not find any association of the analyzed polymorphisms with clinical parameters or the course of the disease. This finding suggests that the proteolytic function of t-PA and its regulation by its major inhibitor is more important for MS onset than its role in fibrinolysis. Because t-PA is biochemically linked to the matrix metalloproteinase (MMP) axis of extracellular proteolysis, it could be important for future studies to examine the relationship between t-PA and MMPs, at the level of the gene-gene interaction or on the level of the functional interaction, which might include the inflammatory cells that infiltrate the brain through the disrupted BBB.

Although the role of fibrinolysis and extracellular proteolysis has been described in both EAE and MS, this study is among the first to analyze the insertion/deletion polymorphisms in PAI-1 and TPA genes in the context of MS. Validation of our results in other populations and larger sample sizes is needed.

## 5. Conclusion

In conclusion, we found a significantly harmful effect of the PAI-5G/5G genotype on MS in both a study cohort and meta-analysis. A more significant effect on MS susceptibility compared to separate gene analysis was shown for the combination of the PAI-1 5G/5G genotype and TPA I allele. As shown for the other gene loci in MS, these results indicate the importance of gene-gene interactions in complex diseases such as MS.

## Figures and Tables

**Table 1:** Characteristics of MS patients.

Parameter	Serbian (*n* = 382)	BH (*n* = 170)	Croatian/Slovenian (*n* = 333)	Pooled samples (*n* = 885)
Gender (female/male)	241/141 (63.1/36.9)	120/50 (70.6/29.4)	239/94 (71.7/28.3)	600/285 (67.8/32.2)
Age of disease onset (years)	29.2 ± 7.0	30.8 ± 8.0	29.4 ± 8.9	30.1 ± 8.0
MSSS	5.4 ± 2.5	4.8 ± 2.8	4.9 ± 2.7	5.2 ± 2.6

MSSS: Multiple Sclerosis Severity Score.

Values are expressed as mean ± SD.

**Table 2:** Frequencies of TPA I/D and PAI-1 4G/5G genotypes and alleles in MS patients and controls and uncorrected ORs for MS susceptibility.

Genotype/allele	Serbian cohort				BH cohort				Pooled samples*			
	MS patients *n* (%)	Controls *n* (%)	OR (95% CI)	*P*	MS patients *n* (%)	Controls *n* (%)	OR (95% CI)	*P*	MS patients *n* (%)	Controls *n* (%)	OR (95% CI)	*P*
TPA I/D												
II	117 (30.6)	98 (30.8)	0.99 (0.72–1.37)	0.957	54 (31.8)	42 (24.7)	1.42 (0.88–2.28)	0.149	277 (31.3)	261 (30.5)	1.04 (0.85–1.27)	0.715
ID	181 (47.4)	134 (42.1)	1.24 (0.92–1.67)	0.165	74 (43.5)	87 (51.2)	0.73 (0.48–1.13)	0.158	408 (46.1)	367 (42.9)	1.14 (0.94–1.38)	0.176
DD	84 (22.0)	86 (27.1)	0.76 (0.54–1.01)	0.121	42 (24.7)	41 (24.1)	1.03 (0.63–1.70)	0.899	200 (22.6)	228 (26.6)	0.80 (0.65–1.00)	0.050
Total	**382**	**318**			**170**	**170**			**885**	**856**		

Allele I *n* (%)	415 (54.3)	330 (51.9)	1.10 (0.89–1.36)	0.364	182 (53.5)	171 (50.3)	1.14 (0.84–1.54)	0.399	962 (54.4)	889 (52.0)	1.10 (0.96–1.26)	0.152
Allele D *n* (%)	349 (45.7)	306 (48.1)	0.91 (0.73–1.12)	0.364	158 (46.5)	169 (49.7)	0.88 (0.65–1.19)	0.399	808 (45.6)	823 (48.0)	0.91 (0.79–1.04)	0.152

PAI 4G/5G												
4G4G	118 (30.9)	88 (27.7)	1.17 (0.84–1.62)	0.353	41 (24.1)	44 (25.9)	0.91 (0.57–1.49)	0.707	245 (27.7)	241 (28.1)	0.98 (0.79–1.20)	0.827
4G5G	182 (47.6)	182 (57.2)	0.68 (0.50–0.92)	0.012	97 (57.1)	87 (51.2)	1.27 (0.83–1.94)	0.277	457 (51.6)	469 (54.8)	0.88 (0.73–1.06)	0.188
5G5G	82 (21.5)	48 (15.1)	1.54 (1.04–2.28)	0.032	32 (18.2)	39 (22.9)	0.78 (0.46–1.32)	0.351	183 (20.7)	146 (17.1)	1.27 (0.97–1.61)	0.054
Total	**382**	**318**			**170**	**170**			**885**	**856**		

Allele 4G *n* (%)	418 (54.7)	358 (56.3)	0.94 (0.76–1.16)	0.554	179 (52.6)	175 (51.5)	1.05 (0.78–1.42)	0.759	947 (53.5)	951 (55.5)	0.92 (0.80–1.05)	0.225
Allele 5G *n* (%)	348 (45.3)	278 (43.7)	1.07 (0.86–1.32)	0.554	161 (47.4)	165 (48.5)	0.95 (0.71–1.29)	0.759	823 (46.5)	761 (44.5)	1.09 (0.95–1.24)	0.225

*Subjects analyzed in the current study (Serbian and BH) and subjects analyzed previously (Croatian and Slovenian) [12].

**Table 3:** The uncorrected odds ratios (ORs) for combination genotypes of the TPA I/D and PAI 4G/5G polymorphisms in MS patients and controls.

Genotype combinations	MS patients (*n* = 885)	Controls (*n* = 856)	OR (95% CI)	*P*
PAI 4G4G/TPA DD	58 (6.6%)	71 (8.3%)	0.78 (0.54–1.1)	0.167
PAI 5G5G/TPA DD	39 (4.4%)	38 (4.4%)	1 (0.63–1.57)	0.974
PAI 4G carrier/TPA DD	161 (18.2%)	185 (21.6%)	0.81 (0.64–1.02)	0.074
PAI 5G5G/TPA I carrier	144 (16.3%)	105 (12.3%)	1.39 (1.06–1.82)	0.017
